# A modified method for precise anastomosis during laparoscopic low anterior resection for rectal cancer: the first clinical experience and application

**DOI:** 10.1186/s12893-024-02335-0

**Published:** 2024-02-09

**Authors:** Bobo Zheng, Ben Wang, Zeyu Li, Yaqi Qu, Jian Qiu

**Affiliations:** Department of General Surgery, Shaanxi Provincial People’s Hospital, Xi’an, Shaanxi China

**Keywords:** Sacral promontory, Distal rectal resection margin, Distance, Laparoscopic low anterior resection, Precision anastomosis

## Abstract

**Background:**

There is no criterion to guide and evaluate the anastomosis of laparoscopic low anterior resection (LAR). We developed a new technique for precise anastomosis. This study endeavored to evaluate the effectiveness and safety of this new technology.

**Methods:**

Patients with mid-low rectal cancer who underwent laparoscopic LAR in our department were enrolled retrospectively between January 1, 2021 and July 1, 2023. During the LAR, the distance between the sacral promontory and the rectal stump was measured and used to determine the length of the sigmoid colon, which was preserved for anastomose. The demographic characteristics and short-term outcomes were analyzed.

**Results:**

Forty-nine patients (26 men, 23 women) with low and middle rectal cancer were retrospectively enrolled in the study. The distance of the tumor from the anal verge was 6.4 ± 2.7 cm. The operative time was 193 ± 42 min. All patients underwent precise anastomosis, among which 12 patients underwent freeing of the splenic flexure of the colon. According to our criteria, there was no redundant or tense state of the colon anterior to the sacrum after the anastomosis. Only one patient had a postoperative anastomotic leak (Grade B). All 15 patients receiving neoadjuvant chemoradiotherapy underwent terminal ileostomy. No postoperative death occurred within 30 days of the surgery. The median follow-up time in our study was 12 months. One patient developed a single metastasis in the right lobe of the liver in the eighth month after surgery and underwent microwave radiofrequency ablation, which did not recur in the four months of postoperative follow-up, and the rest of the patients survived disease-free without recurrence of metastasis.

**Conclusions:**

Precise measurement of the proximal colon of the anastomosis can ensure accurate and convenient colorectal anastomosis and this may be a technique worthy of clinical application. However, its effectiveness needs to be further verified in a multicenter clinical trial.

**Supplementary Information:**

The online version contains supplementary material available at 10.1186/s12893-024-02335-0.

## Introduction

In the 1980s, Heald and colleagues introduced total mesorectal excision (TME), which was a landmark in the history of rectal cancer surgery [1]. Both the National Comprehensive Cancer Network (NCCN) and European Society for Medical Oncology (ESMO) guidelines recommend TME as the golden standard for rectal cancer surgery [2, 3]. In recent years, laparoscopic rectal cancer surgery has been associated with similar long-term outcomes to open surgery, with the benefit of faster postoperative recovery [4]. However, anastomotic leakage is one of the most serious complications following rectal cancer surgery. A pooled analysis of long-term data from COLOR and COLOR II randomized controlled trials showed that anastomotic leakage after rectal cancer surgery significantly increased the local recurrence rate and decreased disease-free survival [5]. The incidence of anastomotic leakage after colorectal cancer surgery varies from 5 to 19% [6−9]. Although many factors, such as nutritional status, advanced age, and complications, may increase the risk of postoperative anastomotic leakage [10], blood supply, stapling strength and quality, and anastomotic tension are key factors for successful anastomosis. Surgeons can determine the blood supply to the anastomosis by observing the arterial pulse, the color of the colon, bleeding at the stump of the colon, or fluorescence imaging techniques [11]. However, in order to avoid anastomotic tension, surgeons can only estimate the proximal resection margin according to their personal experience, and there is no standard operating procedure to guide how to perform a tension-free anastomosis. We have developed a precise measurement technique, for which measuring the distance between the sacral promontory and the distal rectal stump (DPR) as a basis for determining the length of the sigmoid colon which was preserved for anastomose and whether the needs to be dissociated. In this study, we evaluate the effectiveness and safety of this new technology based on short-term outcomes.

## Materials and methods

From January 1, 2021 to July 1, 2023, we retrospectively enrolled consecutively hospitalized patients with low and middle rectal cancer who underwent laparoscopic TME surgery. Patients requiring emergency surgery and those undergoing open low anterior resection (LAR) were excluded. Written informed consent was obtained from patients before enrollment. The study protocol was approved by the Ethics Committee of Shaanxi Provincial People’s Hospital (No: 2023-82).

### Surgical technique

**Fig. 1:**
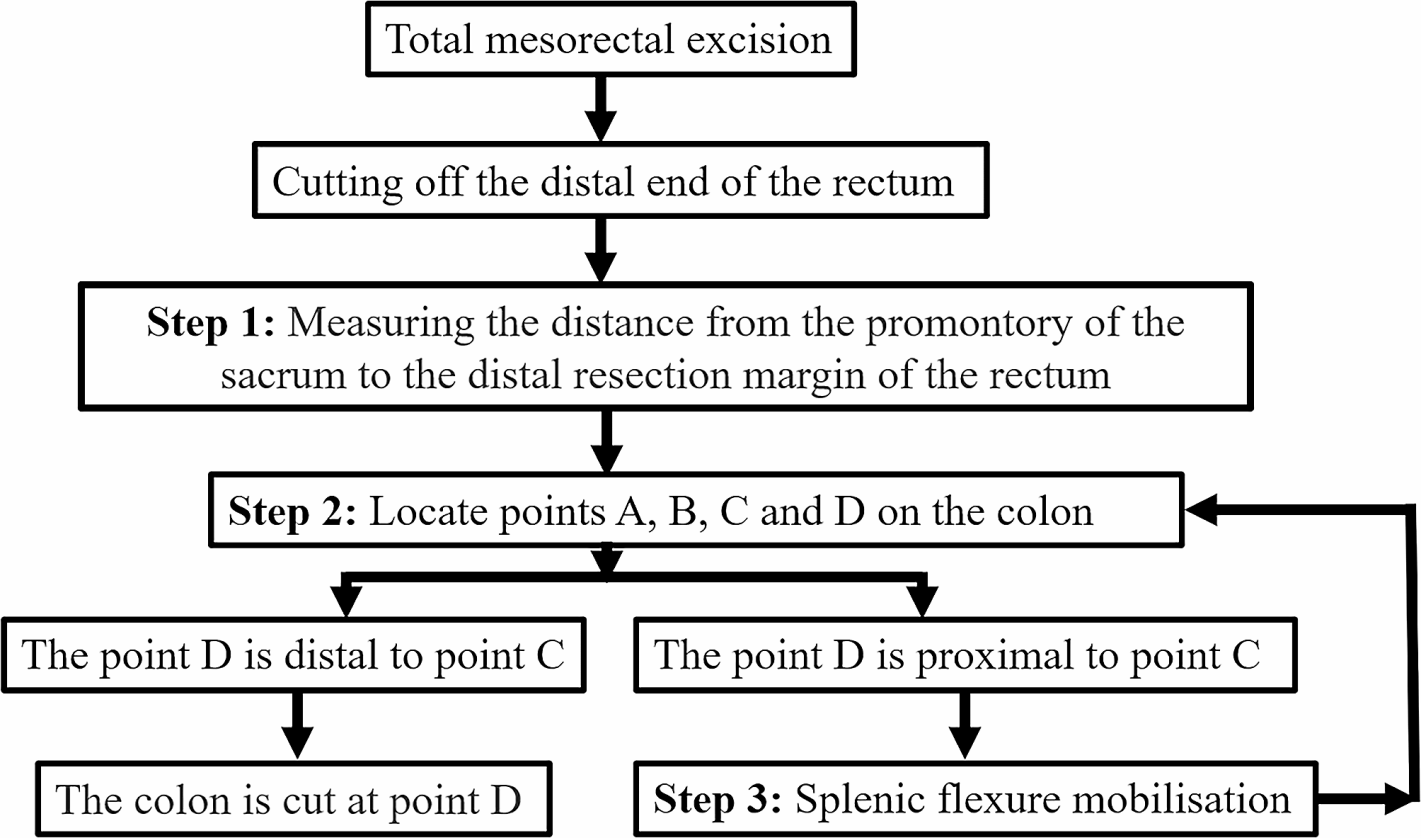
The process of the precise anastomosis technique is shown

All patients underwent laparoscopic TME [12]. The inferior mesenteric artery was ligated proximal to the branch of the left colic artery. The distal resection margin to the low rectal cancer was 2 cm from the lower edge of the tumor. The sacral promontory, caudal to the bifurcation of the abdominal aorta, is defined as the tuberous region protruding from the anterior part of the first sacral vertebra. The process of the precise measurement technique is shown in Fig. 1.

The detailed operating steps are as follows (A video: additional file 1):

- *Step 1*: *Distance measurement*.

**Fig. 2:**
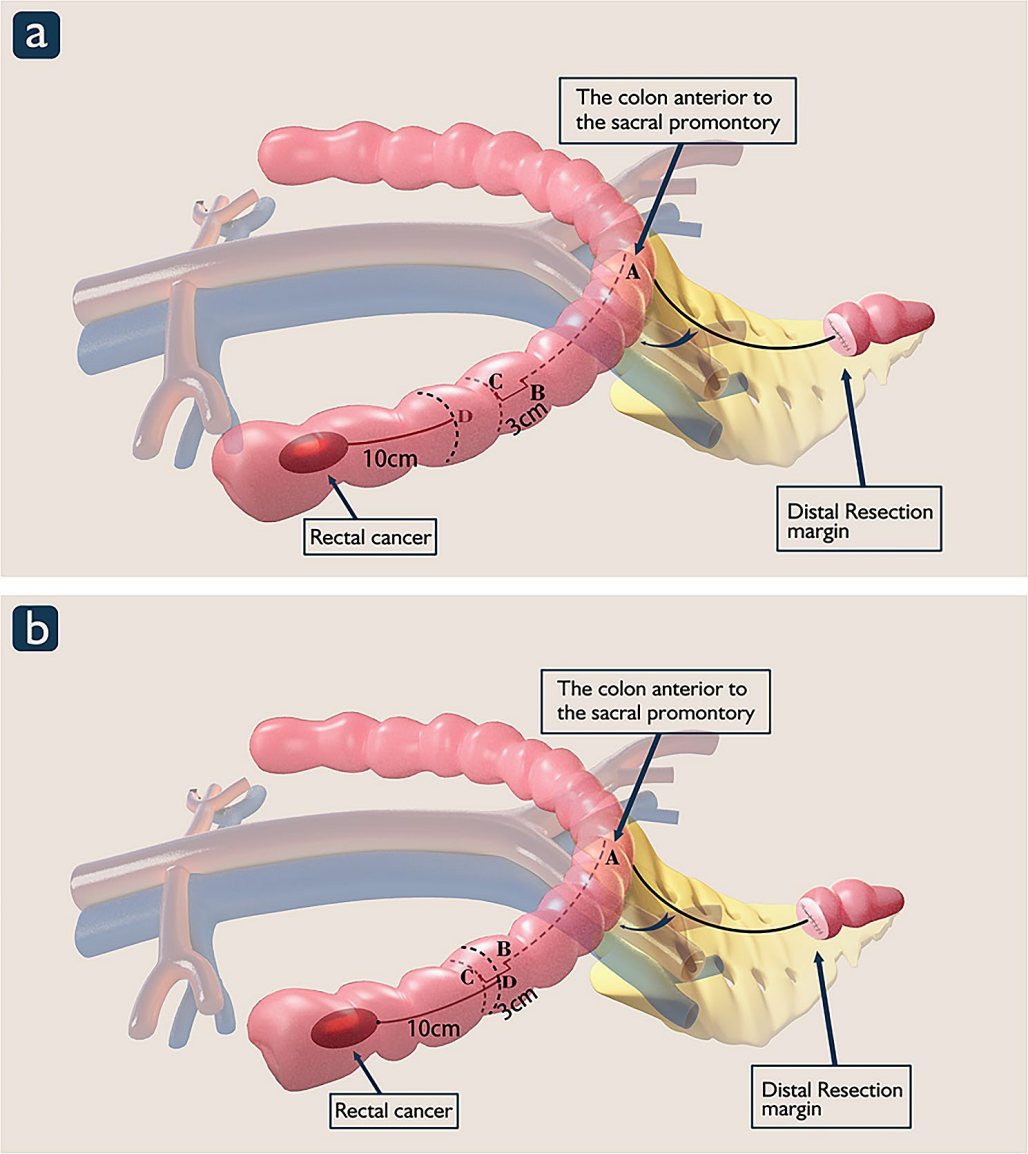
Schematic diagram of precision anastomosis for rectal cancer. Point A is localized in the colon in front of the sacral promontory. Point B: the colon is measured with the cut Mersilk® line distally from the point where the colon is in front of the sacral promontory; the end of the Mersilk® line is marked as point B. Point C: 3 cm is added distally to point B as a pre-excision line for the proximal incision margin, which is marked as point C. Point D:Ten centimeters proximally to the tumor is marked as point D on the colon

**Fig. 3:**
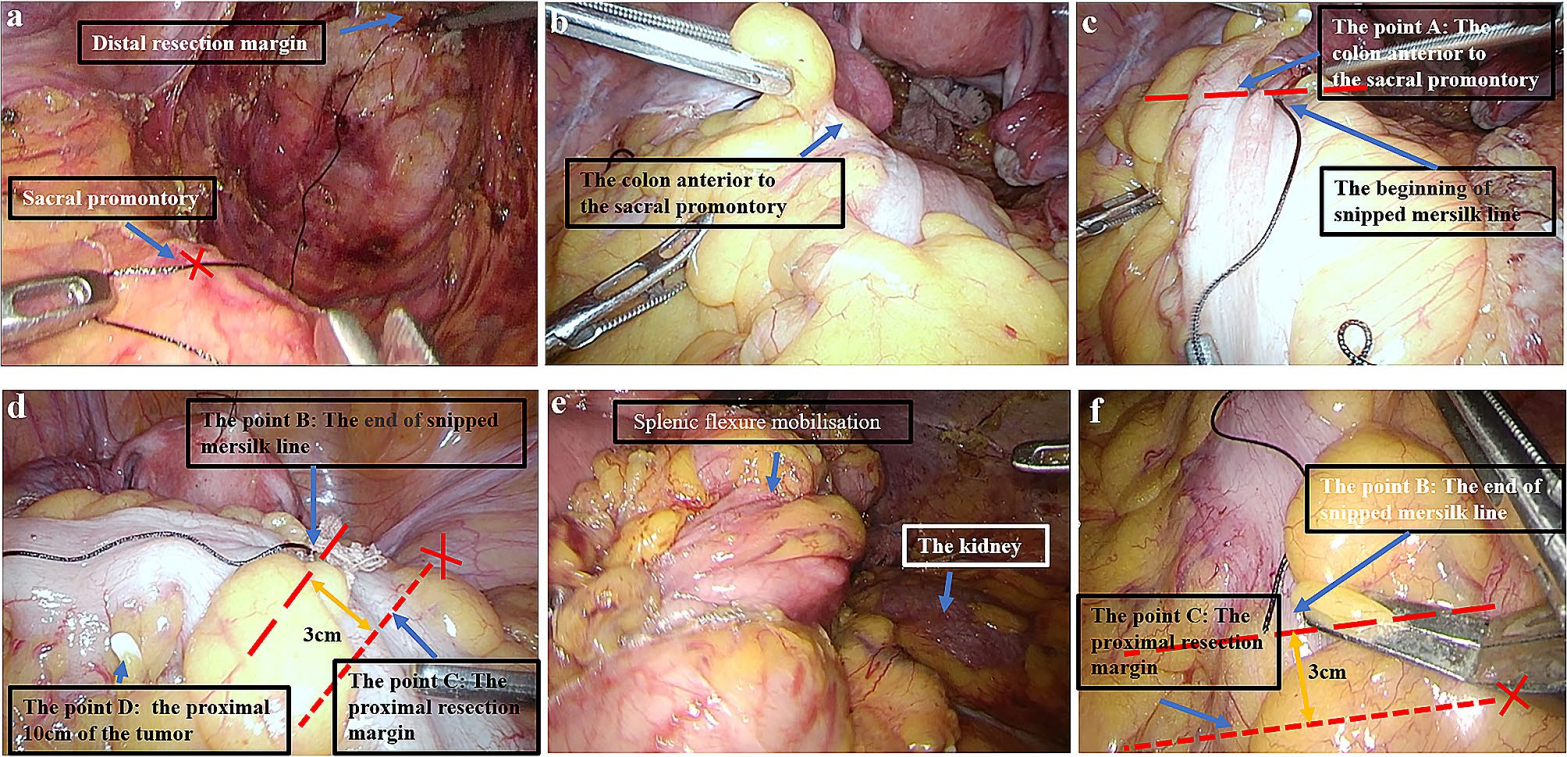
Intraoperative image of precise anastomosis

First, the head of a Mersilk® suture (Ethicon, Johnson & Johnson) was aligned with the stapled end of the DPR. The Mersilk® suture was then placed along the anterior surface of the sacrum until the sacral promontory was reached, where the Mersilk® suture was cut. The length of the Mersilk® suture from the DPR to the sacral promontory was defined as the DPR (Figs. 2 and 3a).

- *Step 2: Determine the proximal resection margin*.

During laparoscopic rectal cancer surgery, the rectum is dissected at 2 cm distal to the tumor, and the left hemicolon is mobilized to meet the required proximal margin and ensure a sufficient length of the colon to the anastomosis. The promontory of the sacrum is referred to as point (A) The left hemicolon is stretched so that it is strung out loosely along the sacral promontory. Then, the colon is measured with the cut Mersilk® line distally from the point where the colon is in front of the sacral promontory; the end of the Mersilk® line is marked as point (B) The 29- or 32-mm circular stapler removes approximately 3 cm of the colon when performing the anastomosis; therefore, 3 cm is added distally to point B as a pre-excision line for the proximal incision margin, *which is marked as point C (*Fig. 2*and* Fig. 3b*−d).*

- *Step 3: Determine whether to free the splenic flexure of the colon*.

Ten centimeters proximally to the tumor is marked as point D on the colon. When point D is distal to point C, there is no need to free the splenic flexure (Fig. 2a). When point D is proximal to point C, freeing of the splenic flexure is needed, in which case point C is subsequentially repositioned (Figs. 2b and 3e−f).

### Evaluation criteria for anastomotic tension

**Fig. 4:**
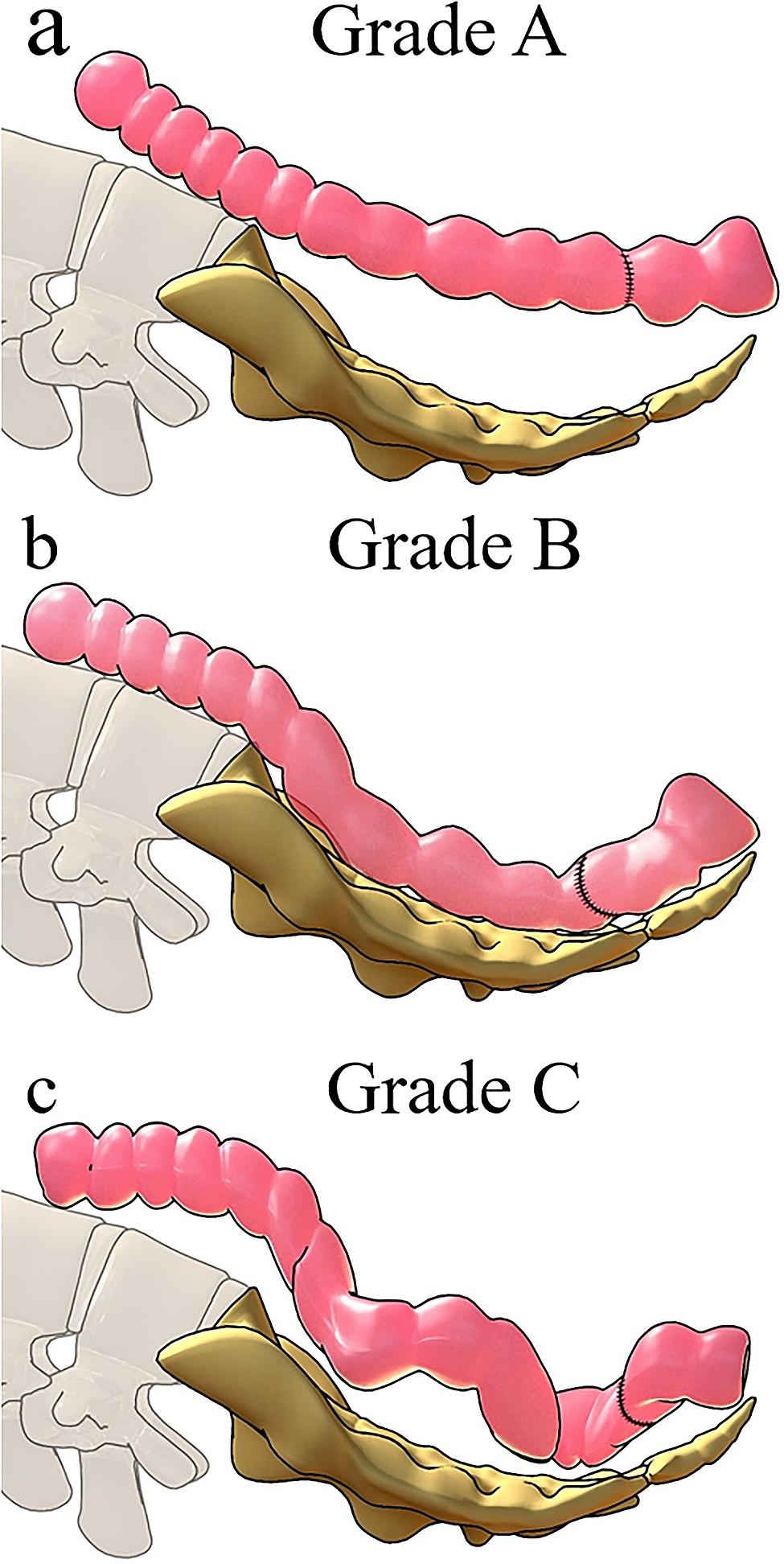
Evaluation criteria for anastomotic tension

Our hospital, through previous surgery experience, has classified the anastomotic tension after laparoscopic LAR of rectal cancer as grade A, B, and C. Grade A represents a colonic overhang anterior to the sacrum after colorectal anastomosis (Fig. 4a); Grade B represents the colon lying flat in front of the sacrum after colorectal anastomosis (Fig. 4b); Grade C represents a long, curved colon lying anterior to the sacrum after colorectal anastomosis (Fig. 4c).

### Statistical analysis

Intraoperative and short-term postoperative outcomes were assessed. Continuous data were presented as mean ± standard deviation. Statistical analysis was performed using SPSS software version 20.0 (IBM, Inc., Armonk, NY, USA).

## Results

Forty-nine patients (26 men, 23 women) with low and middle rectal cancer were retrospectively enrolled in the study (Table 1). The distance of the tumor from the anal verge was 6.4 ± 2.7 cm, and the operative time was 193 ± 42 min. All patients underwent precise anastomosis, among which 12 patients underwent freeing of the splenic flexure of the colon. According to our criteria, there was no redundant or tense state of the colon anterior to the sacrum after the anastomosis. Only one patient had a post-operative anastomotic leak (Grade B). All 15 patients receiving neoadjuvant chemoradiotherapy underwent terminal ileostomy. No postoperative death occurred within 30 days of the surgery (Table 2). We followed up until December 28, 2023, with a median follow-up time of 12 months. One patient developed a single metastasis in the right lobe of the liver in the eighth month after surgery and underwent microwave radiofrequency ablation, which did not recur in the four months of postoperative follow-up, and the rest of the patients survived disease-free without recurrence of metastasis.

**Table 1:** Demographic characteristics

Variables	Value
Sex	
Male	26
Female	23
BMI(Kg/m2)	21 ± 4
Age(year)	62 ± 9
ASA II/III	47/2
The distance of the tumor from the anal margin (cm)	6.4 ± 2.7
Long course neoadjuvant chemoradiotherapy	15
T0-2	34
T3-4	15
N0	33
N1	10
N2	6
Previous surgeries	2
Removal of specimens via natural Orifice(vagina)	4
Removal of specimens via auxiliary incision	35

AJCC, American Joint Committee on Cancer; ASA, American Society of Anesthesiologists; BMI, body mass index

**Table 2:** Outcomes

Outcomes	Value
Operation time(min)	193 ± 42
Hospital stay (days)	6 ± 2
Blood loss (mL)	25 ± 10
Conversion to open surgery	0
Splenic flexure mobilization	12
Anastomotic tension	
Grade A	0
Grade B	49
Grade C	0
The distance between the sacral promontory to the rectal stump (cm)	18 ± 3
the proximal resection margin(cm)	14 ± 2
the distal resection margin(cm)	2 ± 1
No. of lymph nodes	14 (12–18)
Circumferential margin involved	0
Distal margin involved	0
Proximal resection margin positive	0
Macroscopic completeness of resection	
Complete	49
Nearly complete	0
The day of first flatus (days)	3 ± 1
Anastomotic leakage -Grade B	1
Anastomotic bleeding (minimal bleeding)	1
Surgical wound infection	0
Ileus	1
Obstruction	0
Complications associated with ileostomy	0
30-day Mortality	0

✦: After low anterior resection of the rectum, the anastomosis reveals an overhanging and tense colon anterior to the sacrum

*: After low anterior resection, a redundant colon appears anterior to the sacrum after anastomosis due to excessive proximal colon

## Discussion

For a long time, surgeons have focused on total mesenteric resection and lymph node dissection during radical colorectal cancer surgery, and less attention has been paid to gastrointestinal (GI) reconstruction [13]. Therefore, currently, there is no expert consensus or standard on how to precisely reconstruct the GI tract after LAR for rectal cancer. By observing the length of the sigmoid colon and its mesentery, as well as the position of the anastomosis, experienced surgeons are able to estimate whether they need to perform freeing of the splenic flexure of the colon to meet a tension-free anastomosis. However, there are a significant proportion of patients with tension after the anastomosis, in which case, a dangling state of the colon anterior to the sacrum can be observed, which is a high-risk factor for anastomotic leakage [11]. Some surgeons routinely perform freeing of the splenic flexure colon during laparoscopic LAR for rectal cancer. Freeing the splenic flexure of the colon is known to be difficult; thus, it increases the operative time, and not every patient needs it. Moreover, an excessive freeing of the splenic flexure results in redundancy of the anterior sacral colon after anastomosis, which may increase the risk of constipation [14].

The referential anatomical landmark for our precision anastomosis technique is the sacral promontory. The sacral promontory is the most obvious anatomical structure of the sacrum protruding anteriorly, especially when the patients often adopt a modified lithotomy position with the head laying low during the rectal cancer surgery; it is also the marker of the pelvic entrance, so measuring the distance from the sacral promontory to the DPR or cutting edge is easy to be implemented. This technology standardizes precision anastomosis and allows inexperienced colorectal surgeons to achieve precision anastomosis for LAR of the rectum.

With the development of laparoscopic total mesenteric resection for rectal cancer, laparoscopic surgery has become one of the standard procedures for rectal cancer [15]. Laparoscopic or robotic rectal cancer surgery through natural luminal specimen removal has become a candidate procedure for patients with early-stage rectal cancer [16]. These procedures are characterized by GI tract reconstruction, which relies entirely on the operator’s experience. In this context, we propose a precise anastomosis technique that can effectively shorten the operative time, improve surgical fluency, determine the proximal tumor margin with evidence, and provide a technical guarantee for reducing postoperative anastomotic fistula. The incidence of paramedian lymph node metastasis located > 10 cm proximal to rectal cancer tumors has been reported to be 4.5% [17]. Excessive preclusion of the proximal segment of the tumor may increase the risk of tumor recurrence. In this study, the distance of the proximal tumor margin was 14 cm on average, and from the perspective of lymph node dissection, precise anastomosis could help to resect as much proximal colon to the tumor as possible on the basis of safeness, thus reducing the risk of tumor recurrence and metastasis.

The limitations of this study were that the number of cases was not sufficiently large and that no controlled studies were conducted, which may have affected the generalizability of the study to some extent. However, since this technique may compensate to some extent for the shortcomings of relying on experience to perform trimming of the mesentery and free bowel during laparoscopic rectal cancer surgery, it can still give colorectal surgeons, as well as young surgeons, a reliable basis for surgery. Anastomotic leakage can also be influenced by other factors. First of all only two of these cases had a history of previous abdominal surgery, however, since the surgeries were all laparoscopic cholecystectomy histories, there was no impact on the surgery. Second, we have preserved the left colonic artery in all rectal cancer patients, preserving the colonic blood supply to the greatest extent possible, which will minimize the risk of ischemia. Third, prophylactic ileostomy was performed in rectal cancer patients who underwent preoperative neoadjuvant radiotherapy. This also avoided the effect of chemoradiotherapy on anastomotic leakage to a certain extent.

## Conclusion

The precise anastomosis technique based on measuring the distance from the sacral promontory to the distal rectal resection margin holds promise as a safe and effective technique and may lead to standardization of colonic splenic flexure freedom. The technique can provide a reliable standard for surgeons lacking surgical experience and this may be a technique worthy of clinical application. However this study is a summary of our initial clinical experience and has limited ability to draw strong conclusions. Its effectiveness needs to be further verified in multicenter clinical trials.

### Electronic supplementary material

Below is the link to the electronic supplementary material.

Supplementary Material 1

Supplementary Material 2

## Data Availability

No datasets were generated or analysed during the current study.
