# Supplementary material for: A modified method for precise anastomosis during laparoscopic low anterior resection for rectal cancer: the first clinical experience and application
Source: BMC Surg. 2024 Feb 9;24:50. doi: 10.1186/s12893-024-02335-0 (PMC10858553; doi:10.1186/s12893-024-02335-0)
Supplement: Supplementary file 2 — Supplementary Material 2 [file 12893_2024_2335_MOESM2_ESM.docx]

**Legend for the Supplementary video**

After laparoscopic total mesorectal excision, the rectum was isolated approximately 2 cm distal to the tumor, and our modified technique was used in order to achieve a precise anastomosis. This technique is divided into three steps; the first step is to measure the distance from the sacral promontory to the distal cutting edge of the rectum; the second step is to determine points A, B, C, and D, and the third step is to decide whether or not to free the splenic flexure colon through the relationship between points C and D, thus determining the location of the proximal cutting edge of the colon.
